# *Vitis Vinifera* Leaf Extract Protects Against Glutamate-Induced Oxidative Toxicity in HT22 Hippocampal Neuronal Cells and Increases Stress Resistance Properties in *Caenorhabditis Elegans*

**DOI:** 10.3389/fnut.2021.634100

**Published:** 2021-06-11

**Authors:** Chatrawee Duangjan, Panthakarn Rangsinth, Shaoxiong Zhang, Xiaojie Gu, Michael Wink, Tewin Tencomnao

**Affiliations:** ^1^Graduate Program in Clinical Biochemistry and Molecular Medicine, Department of Clinical Chemistry, Faculty of Allied Health Sciences, Chulalongkorn University, Bangkok, Thailand; ^2^Department of Clinical Chemistry, Faculty of Allied Health Sciences, Chulalongkorn University, Bangkok, Thailand; ^3^Leonard Davis School of Gerontology, University of Southern California, Los Angeles, CA, United States; ^4^College of Horticulture, Fujian Agriculture and Forestry University, Fuzhou, China; ^5^Institute of Pharmacy and Molecular Biotechnology, Heidelberg University, Heidelberg, Germany; ^6^Department of Biotechnology, School of Environmental and Chemical Engineering, Dalian Jiaotong University, Dalian, China; ^7^Natural Products for Neuroprotection and Anti-Ageing Research Unit, Department of Clinical Chemistry, Faculty of Allied Health Sciences, Chulalongkorn University, Bangkok, Thailand

**Keywords:** *vitis vinifera*, glutamate toxicity, neuroprotection, HT22, oxidative stress resistanc, daf-16, aging, *caenorhabditis elegans*

## Abstract

*Vitis vinifea* has been used for traditional medicines, food, beverages, and dietary antioxidant supplements. The chemical compositions and biological activities of the fruits and seeds have been extensively investigated. However, the biological effects of the leaves are limited, and its anti-neurodegeneration or antiaging activities are little known. The current work aims to study the beneficial effects of *V. vinifera* leaf extract on neuroprotective effects in HT22 cells, antiaging, and oxidative stress resistance properties in the *Caenorhabditis elegans* model. The ethanol extract was characterized by phytochemical composition using gas/liquid chromatography–mass spectrometry and reversed-phase high-performance liquid chromatography. The beneficial effects of *V. vinifera* ethanol (VVE) extract on antioxidant properties, neuroprotective effects, and the underlying mechanisms were studied by *in vitro* and *in vivo* studies. In HT22 cells, we found that VVE has a protective effect against glutamate-mediated oxidative stress-induced cell death. The gene expression of cellular antioxidant enzymes such as *CAT, SODs, GSTs*, and *GPx* was upregulated by VVE treatment. Moreover, VVE was also shown to alleviate oxidative stress and attenuate reactive oxygen species accumulation in *C. elegans*. We demonstrated that VVE could upregulate the expression of stress-response genes *gst-4* and *sod-3* and downregulate the expression of *hsp-16.2*. Our results suggest that the oxidative stress resistance properties of VVE are possibly involved in *DAF-16*/FoxO transcription factors. VVE reduced age-related markers (lipofuscin) while did not extend the life span of *C. elegans* under normal conditions. This study reports the neuroprotective effect and antioxidant activity of *V. vinifera* leaf extract and suggests its potential as a dietary or alternative supplement to defend against oxidative stress and age-related diseases.

## Graphical Abstract

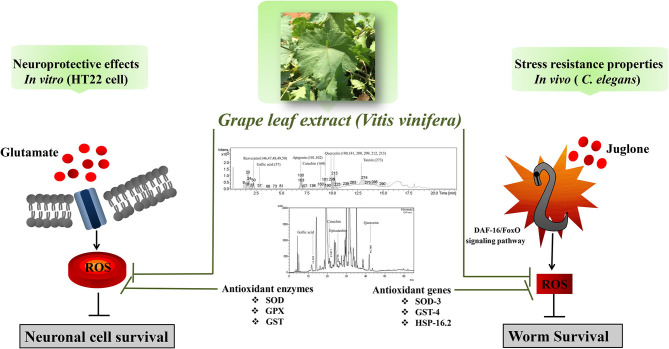

## Introduction

Reactive oxygen species (ROS) imbalance is associated with various neurodegenerative diseases, in particular, Alzheimer's disease (AD) (1, 2). Glutamate is the main excitatory neurotransmitter in the brain, which is considered as one of the initiating factors for neuronal damage (2, 3). A high accumulation of glutamate can cause neuron death *via* accumulated ROS and impaired mitochondrial function (1). The new AD treatment has been focusing on neuroprotection by means of reducing glutamate-induced oxidative toxicity (4). Natural products from herbs or plant extracts that have antioxidant activity and neuroprotective effects could be a potential alternative treatment in neurodegenerative diseases. Herbal compounds have been considered as potential agents for the prevention of AD.

DMSO, dimethyl sulfoxide; LC-MS, gas/liquid chromatography–mass spectrometry; HPLC, high-performance liquid chromatography; ROS, reactive oxygen species; ABTS, 2,2-azino-bis(3-ethylbenzothiazoline-6-sulfonic acid) diammonium salt; DPPH, 2,2-diphenyl-1-picrylhydrazyl; Juglone, 5-hydroxy-1,4-naphthoquinone; SODs, superoxide dismutase; *CAT*, catalase; *GPx*, glutathione peroxidase; *GST*s, glutathione-S-transferase; *SOD-3*, superoxide dismutase-3; *GST-4*, glutathione S-transferase 4; *DAF-16/FoxO*, Forkhead box protein O.

*Vitis vinifera* L. (grape) has been used for food, beverages, and traditional medicine. The leaves have been used in hemorrhoid and diabetic treatments (5). The therapeutic effects are mainly attributed to the phenolic compounds in the fruits, including flavonoids, anthocyanins, and proanthocyanidins (6). These compounds have an antioxidant capacity and antibiotic, antiallergic, antidiarrhea, antiulcer, and anti-inflammatory effects (7, 8). Evidence suggests that the grape seed and skin extracts have a lifespan-extending effect in *C. elegans* (9). The leaf extract of *V. vinifera* has antioxidant and anti-inflammatory activities (10). However, the neuroprotective effects and oxidative stress resistance properties of *V. vinifera* leaf extract in *C. elegans* have not been reported.

In the current study, the neuroprotective effects of *V. vinifera* leaf extract against glutamate-induced cytotoxicity in HT22 cells, oxidative stress resistance properties, and antiaging in *C. elegans* were investigated. This study reports novel neuroprotective effects and antioxidant activity of the *V. vinifera* leaf extract and suggests novel dietary supplements to defend against oxidative stress and age-associated neurodegenerative diseases.

## Materials and Methods

### Chemicals and Reagents

5-Hydroxy-1, 4-naphthoquinone (Juglone) and 2, 7-dichlorofluorescein diacetate were purchased from Sigma-Aldrich GmbH (Steinheim, Germany) and sodium azide from AppliChem GmbH (Darmstadt, Germany). Two, 2-Azino-bis (3-ethylbenzothiazoline-6-sulfonic acid) diammonium salt (ABTS), 2, 2-diphenyl-1-picrylhydrazyl (DPPH), dimethyl sulfoxide (DMSO), Folin–Ciocalteu reagent, L-glutamic acid, quercetin, fetal bovine serum, and Dulbecco's modified Eagle's medium (DMEM) were obtained from Sigma-Aldrich (MO, USA). Gallic acid was purchased from TCI America (OR, USA), 3-(4, 5-Dimethylthiazol-2-yl)-2, 5-diphenyltetrazolium bromide (MTT) from Bio Basic (Ontario, Canada), and Trizol from Invitrogen (CA, USA). Penicillin/Streptomycin solution was purchased from Gibco (MA, USA), CytoTox 96® kit for lactate dehydrogenase (LDH) assay from Promega (WI, USA), reverse transcription (RT) PreMix, and quantitative polymerase chain reaction (qPCR) Master Mix solution from Bioneer (Daejeon, South Korea). The reagents for plant extraction were purchased from RCI Labscan (Bangkok, Thailand).

### Plant Material and Extraction

The leaves of *V. vinifera* were collected from the Pak Chong district, Nakhon Ratchasima Province, Thailand (14.7125°N, 101.421944°E) in July 2016. A voucher specimen of *V. vinifera* (BCU-016295) has been deposited at the herbarium of Kasin Suvatabhandhu, Department of Botany, Faculty of Science, Chulalongkorn University, Thailand.

The leaves of *V. vinifera* were dried at shadow for 1–2 weeks and were grounded into a powder. The powder sample (40 g) was subjected to sequential extraction with solvents of different polarities (hexane, dichloromethane, and ethanol at boiling temperature 70–80°C) by Soxhlet for 36 h (11, 12). The supernatants were combined, subsequently filtered (Whatman No. 1 filter paper), and evaporated at 35–45°C by using a vacuum evaporator. The crude extracts were stored at −20°C as a stock. The residue was dissolved in DMSO to a final concentration of 100 mg/ml as a stock solution before the experiments.

The extraction yields of hexane, dichloromethane, and ethanol fractions were 1.32, 0.35, and 18.93%, respectively. The therapeutic effects of *V.vinifera* products are mainly attributed to the phenolic compounds (6). Ethanol has been frequently used as a solvent for polyphenol extraction and is safe for human consumption (13). Moreover, the ethanol fraction showed the highest yield compared with hexane and dichloromethane fractions. Thus, the *V. vinifera* ethanol extract was used in this study.

### Qualitative Phytochemical Screening

The phytochemical composition of the ethanol extract was analyzed using gas/liquid chromatography–mass spectrometry (LC-MS) (Institute of Systems Biology, University Kebangsaan Malaysia, Malaysia) and reversed-phase high-performance liquid chromatography (HPLC) (RSU Science and Technology Research Equipment Center, Rangsit University, Pathumtani, Thailand) (14) (Supplementary Materials).

### *In vitro* Evaluation of Antioxidant Properties

#### Radical Scavenging Activity

The antioxidant activity of the *V. vinifera* ethanol (VVE) extract was determined by measuring the decrease in the absorbance of the stable free radical ABTS and DPPH, following our methods as described previously (15). Briefly, the DPPH and ABTS were prepared in ethanol at 0.2 mg/ml. The reaction consisted of ABTS or DPPH solution and different concentrations of the VVE extract at a 9:1 ratio. The mixture was incubated in the dark for 30 min at RT. The absorbance values of DPPH and ABTS were measured at 734 nm or 517 nm, respectively, using an EnSpire® Multimode Plate Reader (Perkin-Elmer). The percent inhibition values of the radical and IC50 were calculated as described previously (15). The antioxidant capacity was expressed as vitamin C equivalent antioxidant capacity in milligrams per gram of dry weight plant extract (15).

#### Total Phenolic Content

The assay was carried out according to the Folin–Ciocalteu method and described in our previous work (15). In brief, a Folin–Ciocalteu's phenol reagent (10-fold diluted) and the extract (1 mg/ml) were mixed in a 1:1 ratio and incubated for 20 min. Next, a 7.5% (w/v) Sodium carbonate solution was added to the mixture and kept in the dark at RT for 20 min. The absorbance was read at 760 nm using an EnSpire® Multimode Plate Reader (Perkin-Elmer) as described previously (15). The calibration curve of standard (gallic acid) was used to calculate the total phenolic content, expressed as gallic acid equivalents (GAE.g of plant extracts).

#### Total Flavonoid Content

The assay was carried out according to the aluminum chloride colorimetric method and described in our previous work (15). Briefly, the extract was mixed with 10% (v/v) aluminum chloride solution and 1-M sodium acetate solution, followed by incubating for 40 min in the dark. After that, the absorbance was measured at 415 nm. The calibration curve of standard (quercetin) was used to calculate the total flavonoid content, expressed as quercetin equivalents (QE.g of plant extracts).

### Cell Culture

Mouse hippocampal HT22 cells were obtained from Professor David Schubert (Salk Institute, San Diego, CA, USA) and cultured in DMEM supplemented with 10% fetal bovine serum and 1% penicillin/streptomycin under 5% carbon dioxide at 37°C.

### Cell Treatment

HT22 cells were seeded in tissue culture plates (5,000 cells/well in 96 well-plates, 8,000–10,000 cells/well in 12 well-plates) for 12–18 h. After that, cells were treated with different concentrations of VVE extract (10–100 μg/ml) for 48 h. To induce 40–50% cell toxicity, the culture medium was added with 5-mM glutamate and incubated for 18 h. Stock solutions of glutamate and VVE extract were prepared in DMEM and DMSO, respectively. For the control group, cells were treated with 0.1% (v/v) DMSO.

### Determination of Cell Viability

Cell viability was evaluated by using MTT and LDH assay (Supplementary Materials).

### Measurement of Intracellular Reactive Oxygen Species in HT22 cells

ROS production was quantified by the dichlorofluorescein-diacetate (DCFH-DA) method (Supplementary Materials).

### RNA Isolation and Quantitative Reverse Transcription Polymerase Chain Reaction

Total RNA was extracted using the Trizol reagent (Invitrogen) following the manufacturer's instructions. Reverse transcription was done according to the recommended manufacturers' protocols of AccuPower RT PreMix (Bioneer). The q-PCR was performed in an Exicycler™ 96 (Bioneer). The PCR results were measured using fluorescent signals. The PCR conditions were: 95°C for 15 min, denaturation at 95°C for 15 s for 45–55 cycles, and primer annealing/extension at 55°C for 30 s. The primer specificity test was performed by melting curve. β-actin (internal control gene) was used to normalize the relative expression levels by using the 2^−ΔΔCT^ method. The gene-specific sequences of primers were *CAT, SOD1, GPx, GSTo1, GSTa2*, and β*-actin* (3) (Supplementary Materials).

### *C. elegans* Strains and Culture Conditions

The strains N2 (wild type), TJ375 [gpIs1(*hsp*-16-2::GFP)], TJ356 [zIs356 (daf-16p::daf-16a/b::GFP+rol-6)], CF1553 {muls84[pAD76(*sod*-3::GFP)]}, CF1038 [daf-16(mu86)I], BA17 [fem-1(hc17)IV], CL2166 [(pAF15)gst-4p::GFP::NLS], and *Escherichia coli* OP50 were obtained from the Caenorhabditis Genetics Center at the University of Minnesota, USA. All strains were maintained at 20°C and cultured on nematode growth media (NGM) plates with living *E. coli* OP50. For all assays, the larvae (L1 stage) were seeded in liquid medium (S-medium), inoculated with *E. coli* OP50. Synchronous populations were obtained by using hypochlorite treatment (5-M sodium hydroxide and 5% sodium hypochlorite). The eggs were allowed to hatch in M9 buffer as described previously (15, 16). For the treatment groups, worms were treated with different concentrations of VVE extract: 25, 50, and 100 μg/ml. For the control group, worms were treated with 0.1% (v/v) DMSO.

### Survival Assay Under Juglone-Induced Oxidative Stress

L1 larvae of wild-type (N2) and CF1038 transgenic strains were treated with different concentrations of VVE extract in S-medium for 48 h. After treatment, worms were exposed to the pro-oxidant juglone at 80 μM for 24 h. The dead and live worms were counted by gentle touch with a platinum wire.

### Measurement of Intracellular Reactive Oxygen Species in *C. elegans*

L1 larvae of wild-type (N2) and CF1038 transgenic strains were treated with different concentrations of VVE extract in S-medium for 48 h. After treatment, ROS production was quantified by the DCFH-DA method according to our previous work (15, 17). The 50-μM DCFH-DA was added into S-medium and incubated in the dark at 20°C for 1 h.

Worm images were examined under a fluorescent microscope (Keyence Deutschland GmbH, Neu-Isenburg, Germany) at least 30 worms per group. The relative fluorescence of the whole body was examined using ImageJ software (National Institutes of Health, Bethesda, MD). The results are presented as mean fluorescence ± SEM.

### Quantification of *hsp-16.2* Expression

L1 larvae of TJ375 transgenic worms, which carry *hsp-16.2* promoter regions fused with a green fluorescent protein (GFP) reporter, were treated with different concentrations of VVE extract in S-medium at 20°C for 72 h. Then, the worms were induced by exposing a nonlethal dose of 20-μM juglone for 24 h. After incubation, worms were anesthetized by the addition of 10-mM sodium azide. Then, worms were mounted on a microscopic glass slide. The expression of *hsp-16.2* was examined by observing the fluorescence at the anterior part from the back of the pharynx as described previously (15, 18).

### Quantification of *sod-3* Expression

L1 larvae of CF1553 transgenic worms, which carry *sod-3* promoter regions fused with a GFP reporter, were treated with different concentrations of VVE extract in S-medium at 20°C for 72 h. Then, worms were submitted to fluorescence microscopy as described previously (15, 18).

### Quantification of *gst-4* Expression

L1 larvae of CL2166 transgenic worms, which carry *gst-4* promoter regions fused with a GFP reporter, were treated with different concentrations of VVE extract in S-medium at 20°C for 48 h. Next, the worms were induced by exposing a nonlethal dose of 20-μM juglone for 24 h. Fluorescence images were taken by fluorescence microscopy as described previously (15, 18).

### Determination of Subcellular Localization of DAF-16

L1 larvae of TJ356 transgenic worms were treated with different concentrations of VVE extract in S-medium for 48 h and submitted to fluorescence microscopy as described previously (15, 18).

### Assessment of Autofluorescent Pigment

BA17 transgenic worms were used for measuring the accumulation level of the autofluorescent pigment lipofuscin. L1 larvae of BA17 transgenic worms were treated with different concentrations of VVE extract in S-medium and maintained at 25°C to prevent egg-laying. The media was changed every second day. On day 16, the worms were anesthetized by the addition of 10-mM sodium azide, mounted on a glass slide, and photographed. Worms were photographed using a BIOREVO BZ-9000 fluorescence microscope (λex 360/20 nm, λem 460/38 nm) as described previously (15).

### Longevity Assay

The wild-type (N2) worms were used for the lifespan assay under normal conditions. Synchronization and treatment were conducted as described previously (15). In brief, N2 worms were synchronized at the L4 larval stage on NGM agar plates supplemented with VVE extracts and *E. coli* OP50 at 20°C. The treatment plate was prepared by mixing VVE extracts (final concentration 50 μg/ml) with *E. coli* OP50 and adding on NGM agar plate overnight before use. The worms were counted during the transfer to fresh medium every day. After that, the percentages of surviving worms were documented. Worms that failed to respond to a gentle touch with a platinum wire were scored as dead and excluded from the plates. Internal hatched progeny worms were scored as censors and discarded from the assay.

### Brood Size and Body Length Assay

To analyze the potential toxic effect of the extract on the reproductive system, brood size was measured as described in our previous work (15, 18) (Supplementary Materials).

### Statistical Analysis

In these studies, the results are presented as the mean ± SEM and were analyzed with GraphPad Prism 8. The experiments were performed in at least triplicate. One-way analysis of variance (ANOVA) following Bonferroni's method (*post hoc*) analyzed a comparison between the control and treatments. Differences were considered significant at the *P* ≤ 0.05 level.

## Results and Discussion

### Phytochemical Constituents of *V. vinifera* Ethanol Extract

**Figure 1:**
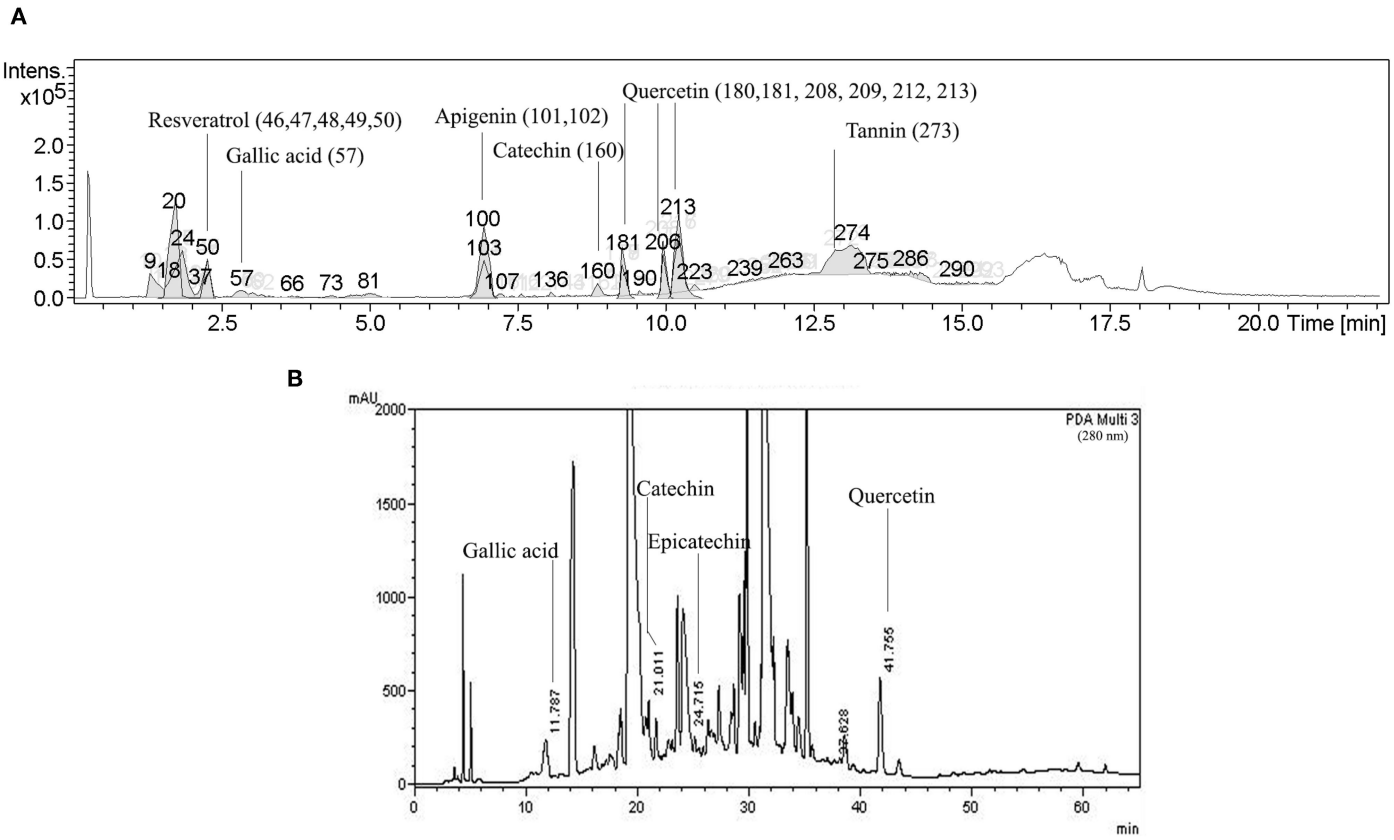
Representative bioactive compounds in VVE extract. LC-MS-MS **(A)** and HPLC **(B)** profiles of major compounds in VVE extract.

In this study, LC-MS and HPLC were carried out for the tentative identification of the phytoconstituents in the VVE extract. A phytochemical profile is shown in Figure 1. The detected and identified compounds are listed in Supplementary Table 1 with the corresponding retention and MS/MS fragmentation data.

We tentatively identified the main compounds in the VVE extract, including resveratrol, gallic acid, apigenin, catechin, quercetin, and tannin. Fingerprinting analysis of VVE extracts using HPLC showed the presence of the bioactive compound gallic acid (18.26 mg/100 g of crude extract), catechin (55.10 mg/100 g of crude extract), epicatechin (14.22 mg/100 g of crude extract), and quercetin (197.73 mg/100 g of crude extract) (Supplementary Figure 1B, Table 2). Our results thus agree with the published chemical composition of *V. vinifera* leaf extracts (6, 10).

## *In vitro* Studies

### Effect of *V. vinifera* Ethanol Extract on Glutamate-Induced Cytotoxicity in HT22 Cells

Excessive glutamate induced oxidative stress leading to neurotoxicity and neuronal cell death (2). The immortalized mouse hippocampal HT22 cells are common cell models to evaluate glutamate toxicity caused by oxidative stress. These cells lack ionotropic glutamate receptors, which exclude excitotoxicity as a cause of glutamate-triggered cell death (2).

**Figure 2:**
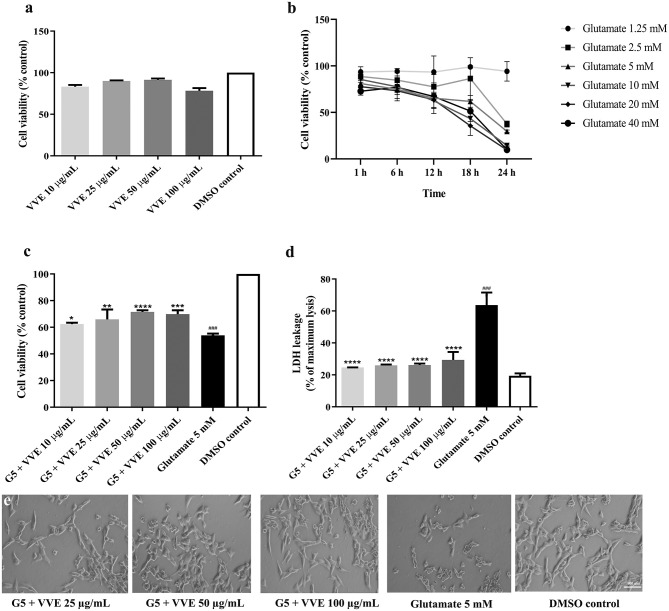
Protective effects of VVE extracts against glutamate-induced toxicity in HT22 cells. Cell viability by treatment with different concentrations of VVE extracts for 48 h **(a)** and cell viability by treatment with different concentrations of glutamate for different times **(b)**. Cells were treated with different concentrations of VVE extracts for 48 h and exposed to 5-mM glutamate for 18 h. Then, cell viability was measured by MTT **(c)** and LDH **(d)** assay. Cell morphology was observed under a microscope at 5 × magnification **(e)**. Samples were exposed to 5-mM glutamate (**g**) to induce toxicity. All data are shown as mean ± SEM of at least three independent experiments. ^####^*p* < 0.0001 vs. DMSO control; *p < 0.05, **p < 0.01, ***p < 0.001, and ****p < 0.0001, compared with glutamate-treated cells by one-way ANOVA following Bonferroni's method (*post hoc*).

To investigate whether the VVE extract could prevent cell death induced by glutamate, the protective effects against glutamate-induced oxidative toxicity were explored in HT22 cells using MTT, LDH assay, and cell morphological examination. First, we determined the non-cytotoxic concentration of the extract and the optimum condition of glutamate in HT22 cells. We found that the VVE extract was relatively non-cytotoxic at the tested doses (10–100 μg/ml VVE, 48 h) (Figure 2a), and the cell viability was reduced by approximately 50% at the tested doses (5-mM glutamate, 18 h) (53.9 ± 0.6% (*p* < 0.001) (Figure 2b).

Surprisingly, the viability of the HT22 cells pretreated with VVE extract had significantly lower glutamate-induced cell death compared with that of the cells exposed to glutamate alone [(Figures 2c–e) 10, 25, 50, and 100 μg/ml VVE-induced survival rate against glutamate-induced cell death by 8.4 ± 0.6% (*p* < 0.05), 11.9 ± 4.3% (*p* < 0.01), 17.6 ± 0.7% (*p* < 0.0001), and 14.9 ± 1.7% (*p* < 0.001), respectively]. The results suggest that VVE extract exerts a potent neuroprotective effect against glutamate-induced cytotoxicity in HT22 cells.

### Effect of *V. vinifera* Ethanol Extract on Glutamate-Induced Oxidative Stress in HT22 Cells

**Figure 3:**
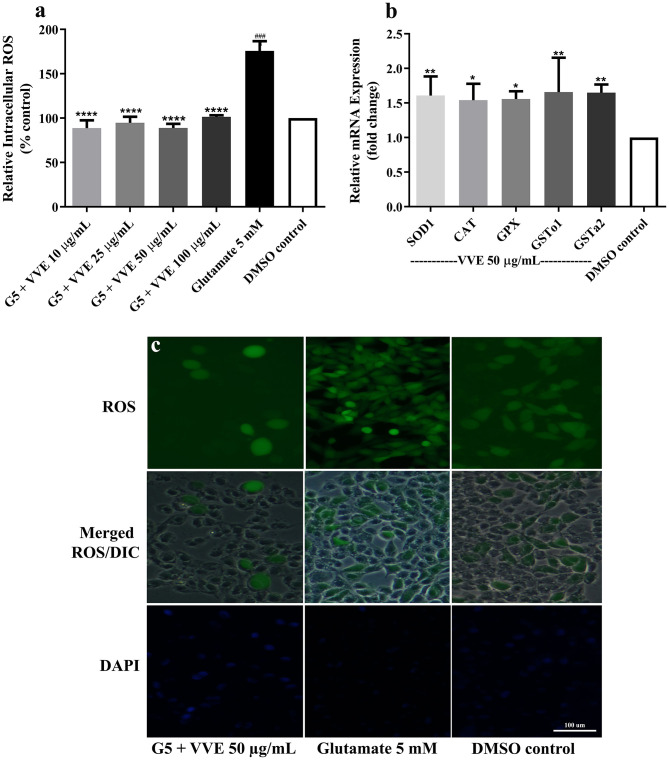
Protective effect of VVE extracts against glutamate-induced oxidative stress in HT22 cells. VVE extracts treatment reduced ROS levels in HT22 cells when compared with glutamate-treated cells **(a)**. VVE extract treatment increased endogenous antioxidant gene expression in HT22 cells when compared with DMSO control **(b)**. Samples were pretreatment with VVE extracts for 48 h and exposed to 5-mM glutamate (G5) for 12 h to induce oxidative stress. Representative fluorescence micrographs of cells stained with H_2_DCFDA were observed under a fluorescence microscope **(c)**. All data are shown as mean ± SEM of at least three independent experiments. *p < 0.05, **p < 0.01, ***p < 0.001, and ****p < 0.0001, compared with glutamate-treated cells; ^###^*p* < 0.001, compared with DMSO control by one-way ANOVA following Bonferroni's method (*post hoc*).

Oxidative stress mediates glutamate-induced neuronal cell death, which plays an essential role in neurodegenerative diseases (2). To investigate whether VVE extract could suppress glutamate-induced oxidative stress, we illustrated the antioxidant properties of VVE extract *in vitro* and in cells. The VVE extract showed powerful antioxidant activity *in vitro* with high phenolic and flavonoid contents (Supplementary Figure 1, Table 3). Moreover, the elevated levels of intracellular ROS induced by glutamate were attenuated in the cells pretreated with VVE extract (Figures 3a,c) [10, 25, 50, and 100 μg/ml VVE reduced intracellular ROS levels by 87.1 ± 5.1% (*p* < 0.0001), 81.1 ± 3.9% (*p* < 0.0001), 86.9 ± 2.7% (*p* < 0.0001), and 74.4 ± 1.4% (*p* < 0.0001), respectively].

Both antioxidant properties of VVE extract *in vitro* and in cells suggest that VVE extract protects against glutamate-induced cytotoxicity by inhibiting the accumulation of intracellular ROS. Previous research has indicated that an antioxidant, such as phenolic and flavonoids, strongly prevented ROS-induced neuronal cell death (19). Neuroprotective properties of resveratrol (20, 21), gallic acid (22), apigenin (21), catechin (23), and quercetin (24) were also highlighted in several recent studies. Our results agreed with literature data indicating that the phenolic compounds (resveratrol, gallic acid, apigenin, catechin, quercetin, and tannin) in VVE extract may mediate antioxidant activity and neuroprotective effects in HT22 cells.

### Effect of *V. vinifera* Ethanol Extract on Gene Expression of Antioxidant Enzymes in HT22 Cells

The antioxidant and phase II enzymes, including superoxide dismutase (*SOD*), catalase (*CAT*), glutathione peroxidase (*GPx*), and glutathione-S-transferase (*GST*), have been known as a central role of ROS-mediated cellular damage prevention (3). To further examine the mechanism of antioxidant-mediated neuroprotective effects of the VVE extract, we examined the effects of the VVE extract on antioxidant enzyme (*SOD, CAT, GPx*, and *GST*) gene expression. We found that VVE extract (50 μg/ml) significantly upregulated the expression of endogenous antioxidant enzymes, including *SOD1, CAT, GPx, GSTo1*, and *GSTa2* (Figure 3b).

Our results are in agreement with other studies where grape leaf extracts (*V. vinifera*) were found to protect against oxidative damage by promoting antioxidant gene response in several models, including neuronal cells (25), *C. elegans* (26), and rodents (25, 27, 28). In accordance with previous studies, the bioactive compounds in grape leaf extracts such as resveratrol (29), catechin (29), gallic acid (22), and quercetin (30) also increased antioxidant gene expression.

In the brain, an imbalance of ROS homeostasis is involved in the pathogenesis of several neurodegenerative events (31). Antioxidant balance inside the cells requires intrinsic (endogenous enzymes) and extrinsic (dietary supplements) antioxidants for neutralizing ROS. Natural plants with antioxidant properties have been recognized as precious sources for drug discovery in age-related diseases (24, 32–35). The current results demonstrated that the protective effect of VVE extract against glutamate-induced cytotoxicity is not only through suppressing intracellular ROS production but also through enhancing endogenous antioxidant and phase II enzymes in neuronal HT22 cells.

## *In vivo* Studies

### Effect of *V. vinifera* Ethanol Extract on Juglone-Induced Oxidative Stress in *C. elegans*

*C. elegans* is a valuable model for aging research in studying genetic and pharmacological influences of ROS (36, 37). To further elucidate the antioxidant activities of the VVE extract *in vivo*, the oxidative resistance properties of the VVE extract were conducted in a *C. elegans* model. We first determined the survival of nematodes under oxidative stress conditions.

**Figure 4:**
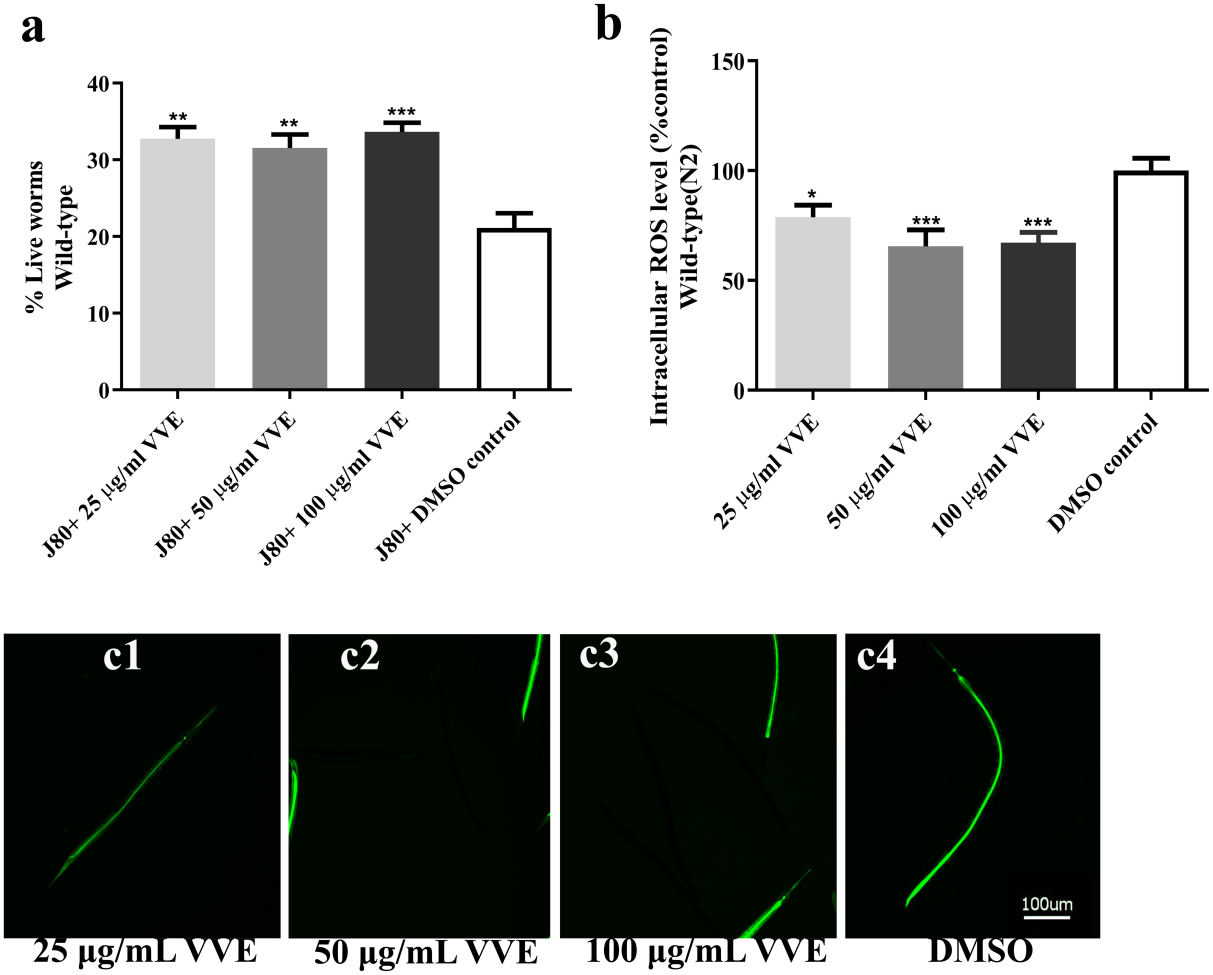
Protective effect of VVE extracts against juglone-induced oxidative stress in *C. elegans*. VVE extracts protect against oxidative stress in wild-type *C. elegans*. Survival rate of wild-type (N2) worms was significantly enhanced after pretreatment with extracts **(a)**. VVE extracts treatment reduced ROS levels in N2 worms when compared with DMSO control **(b)**. Representative pictures of DCFDA fluorescence in wild-type (N2) worms treated with 25 μg/ml VVE **(c1)**; 50 μg/ml VVE **(c2)**; 100 μg/ml VVE **(c3)**; and DMSO control **(c4)**. In survival assay, samples were exposed to 80-μM juglone (J) to induce oxidative stress. All data are shown as mean ± SEM of at least three independent experiments.*p < 0.05, **p < 0.01, ***p < 0.001, and ****p < 0.0001, compared with DMSO control by one-way ANOVA following Bonferroni's method (*post hoc*).

Treatment with different concentrations of VVE extract (25–100 μg/ml) caused no significant changes in the survival rate of wild-type worms compared with the DMSO control (Supplementary Figure 2). However, under oxidative stress conditions (80-μM juglone for 24 h), the survival rate of the wild-type worms pretreated with the VVE extract was significantly increased when compared with the DMSO control (21.1 ± 1.9%) (Figure 4a) [25, 50, and 100 μg/ml VVE reduced mortality by 32.8 ± 1.5% (*p* < 0.01), 31.5 ± 1.8% (*p* < 0.01) and 33.6 ± 1.2% (*p* < 0.001), respectively]. Stress resistance properties are closely related to antioxidant activity (31). Although VVE extracts improved the survival rate of the wild-type worms, compared with control, the survival rate did not improve in a similar range as the epigallocatechin gallate, which is a powerful antioxidant in green tea (38). Similarly, VVE extract exhibited lower scavenging activity than epigallocatechin gallate (Supplementary Figure 2). These results suggest that the antioxidant activity of VVE extract might be partially attributed to improving the survival rate.

Subsequently, the intracellular ROS accumulation was measured to confirm the antioxidant properties of the VVE extract. The ROS indicator DCFH-DA was used to determine the accumulation of ROS levels in wild-type worms. The fluorescence intensity is correlated with the intracellular ROS level. The intracellular ROS accumulations were significantly reduced in the wild- type worms treated with VVE extract groups [25, 50, and 100 μg/ml VVE reduced intracellular ROS accumulation by 21.2 ± 5.5% (*p* < 0.05), 34.4 ± 7.5% (*p* < 0.001), and 32.8 ± 4.8% (*p* < 0.001), respectively] (Figure 4b) (Representative microscopy images from individual worms can be found in Figure 4c). Interestingly, under oxidative stress conditions (a nonlethal dose of 20-μM juglone), VVE extracts also reduced intracellular ROS level in wild-type worms when compared with the worms exposed to juglone alone [25, 50, and 100 μg/ml VVE reduced intracellular ROS accumulation by 74.1 ± 4.6% (*p* < 0.0001), 75.2 ± 5.2% (*p* < 0.0001), and 80.3 ± 4.8% (*p* < 0.0001), respectively] (Supplementary Figure 3A).

However, we found that the lower concentrations of VVE extract (<10 μg/ml in HT22 cells and 25 μg/ml in worms) neither decreased intracellular ROS accumulation level nor increased survival rate under oxidative stress condition compared with the DMSO control (Supplementary Figure 4). These results suggest that the VVE extracts at moderate concentrations have antioxidant activities.

Previous works have reported that grape seed and skin extracts have antioxidants (26) and lifespan-extending effects in *C. elegans* (9). The data support our assumption that the VVE leaf extract contains polyphenols (resveratrol, gallic acid, apigenin, catechin, quercetin, and tannin), which have protective effects against oxidative stress to reduce endogenous ROS levels in *C. elegans*.

### Effect of *V. vinifera* Ethanol Extract on Stress Resistance Properties Mediated by the *DAF-16/FoxO* Pathway in *C. elegans*

DAF-16, the *C. elegans* homolog of the mammalian FOXO transcription factor, is the main transcription factor involved in stress response, metabolism, and longevity (39). *DAF-16/FoxO* remains inactive in the cytosol under normal conditions. In contrast, stress or specific ligands can stimulate its translocation to the nucleus, influencing stress response genes expression such as *hsp-16.2, sod-3*, and *gst-4* (39).

**Figure 5:**
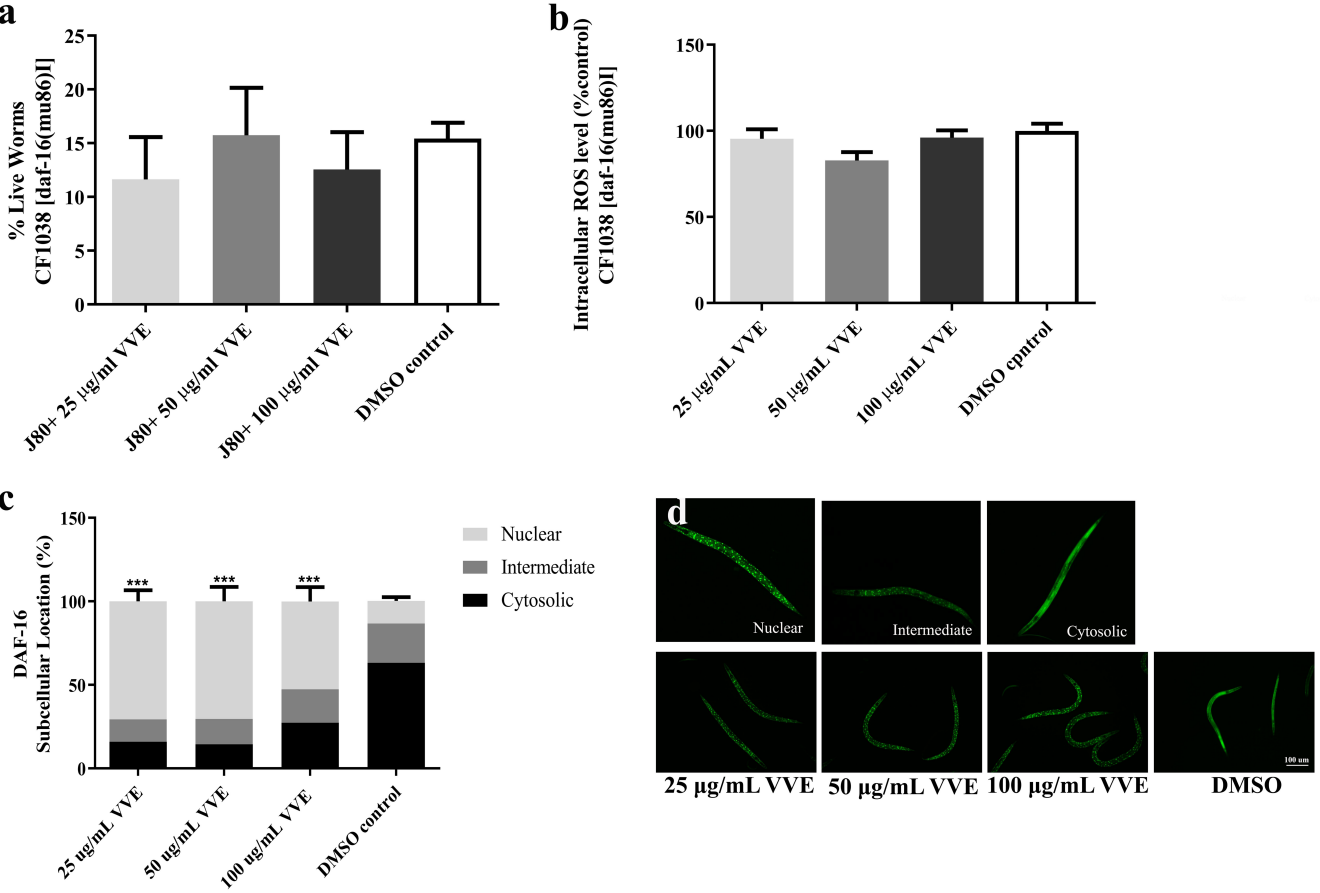
Stress resistance properties of VVE extracts mediated *DAF-16/FoxO* pathway in *C. elegans*. VVE extracts failed to increase survival rate **(a)** and decrease ROS levels **(b)** in CF1038 worms. Moreover, VVE extracts induced a significant translocation of DAF-16::GFP in mutant TJ356 worms (daf-16p::daf-16a/b::GFP + rol-6) **(c)**. Representative fluorescent images of subcellular location of DAF-16 in nucleus, intermediate, cytosolic regions, and TJ356 worms after treated with VVE extracts **(d)**. All data are shown as mean ± SEM of at least three independent experiments.*p < 0.05, **p < 0.01, ***p < 0.001, and ****p < 0.0001, compared with DMSO control by one-way ANOVA following Bonferroni's method (*post hoc*).

To examine the influence of VVE extract on DAF-16 nuclear translocation, DAF-16 transgenic (TJ356) (DAF-16::GFP) worms were used. The majority of the worms treated with DMSO control showed a cytosolic DAF-16::GFP localization (63.3 ± 7.3% cytosolic, 23.5 ± 7.7% intermediate, and 13.6 ± 4.9% nuclear). However, VVE extract significantly increased the level of nuclear location of DAF-16::GFP when compared with the DMSO control [25, 50, and 100 μg/ml VVE induced DAF16::GFP nuclear location by 65.6 ± 6.4% (*p* < 0.001), 63.2 ± 7.4% (*p* < 0.001), and 52.5 ± 8.5% (*p* < 0.001), respectively]. (Figure 5c) (Representative microscopy images from individual worms can be found in Figure 5d).

In oxidative stress conditions, such as juglone treatment, DAF16 has induced translocation into the nucleus (40). We found that the juglone treatment group significantly increased the level of nuclear location of DAF-16::GFP when compared with the untreated control group [by 43.6 ± 7.4% (*p* < 0.0001)] (Supplementary Figure 3B). However, VVE extract blocked the juglone-induced nuclear translocation of DAF-16, suggesting that VVE extract indeed prevented the juglone-induced oxidative stress in *C elegans*.

To further investigate the effects of VVE extract that mediates antioxidant activity through the DAF-16/FoxO pathway, the transgenic CF1038 worms, which are the DAF-16 loss-of-function mutant, were used in survival (Figure 5a) and intracellular ROS accumulation assay (Figure 5b). Interestingly, VVE extract failed to increase the survival rate under oxidative stress (Figure 5a) and attenuate intracellular ROS levels (Figure 5b) in CF1038 worms. The data indicate that VVE extract has antioxidant activity and stress resistance in *C. elegans* through the *DAF-16/FoxO* pathway.

### Effect of *V. vinifera* Ethanol Extract on Gene Expression of Stress Response (*hsp-16.2*::GFP, *sod-3*::GFP, and *gst-4*::GFP) in *C. elegans*

**Figure 6:**
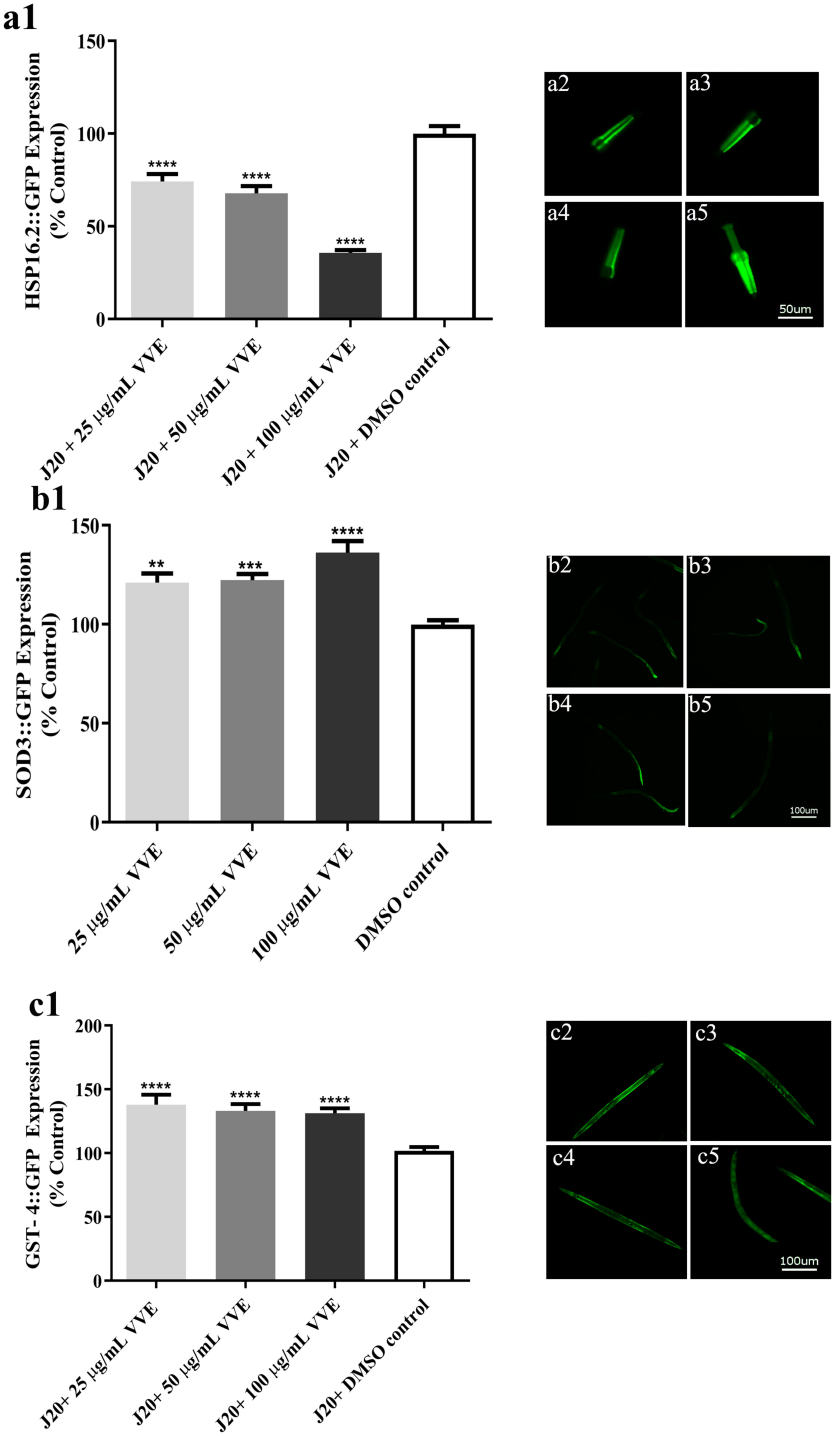
Effect of VVE extracts on expression of stress resistance-related genes in *C. elegans*. VVE extracts decreased *hsp-16.2* expression in mutant TJ375 worms **(a)**, increased *sod-3* expression in mutants CF1553 worms **(b)**, and *gst-4* expression in mutants CL2166 worms **(c)**. a2–a5, b2–b5, c2–c5: Representative pictures of GFP fluorescence in worms treated with 25 μg/ml VVE (a2/b2/c2); 50 μg/ml VVE (a3/b3/c3); 100 μg/ml VVE (a4/b4/c4); and DMSO control (a5/b5/c5). TJ375 and CL2166 worms were exposed to 20-μM juglone to induce mild oxidative stress. *p < 0.05, **p < 0.01, ***p < 0.001, and ****p < 0.0001, compared with DMSO control by one-way ANOVA following Bonferroni's method (*post hoc*).

More evidence that VVE extract can attenuate oxidative stress was obtained by measuring the expression of *DAF-16/FoxO* downstream genes (*hsp-16.2, sod-3*, and *gst-4*). Heat-shock proteins have been known as a sensor of oxidative stress function induced by oxidative stress and heat shock conditions (39). Under mild oxidative stress conditions (20-μM juglone), the head of the transgenic worms (TJ375) exhibited high-intensity GFP fluorescence representative *hsp-16.2* gene induction. However, VVE extract significantly reduced the level of the fluorescence intensity of *hsp-16.2*::GFP when compared with the DMSO control [25, 50, and 100 μg/ml VVE reduced expression level of *hsp-16.2* by 25.9 ± 4.5% (*p* < 0.0001), 32.4 ± 3.8% (*p* < 0.0001), and 64.3 ± 1.4% (*p* < 0.0001), respectively] (Figure 6a).

We further investigate the antioxidant properties of the extract by determining the expression of antioxidant enzymes including *SOD-3* (superoxide dismutase 3) and *GST-4* (glutathione S-transferase 4). We found that the VVE extract significantly increased the level of the fluorescence intensity of *sod3*::GFP (Figure 6b) and *gst-4*::GFP (Figure 6c), in transgenic worms CF1553 and CL2166 worms, respectively [*sod3*::GFP; 25, 50, and 100 μg/ml VVE-induced expression level of *Sod-3* by 21.1 ± 4.6% (*p* < 0.01), 22.3 ± 3.2% (*p* < 0.001), and 36.1 ± 5.9% (*p* < 0.0001), respectively] [*gst-4*::GFP; 25, 50, and 100 μg/ml VVE-induced expression level of *gst-4* by 38.0 ± 7.7% (*p* < 0.0001), 33.1 ± 5.3% (*p* < 0.0001), and 31.3 ± 3.8% (*p* < 0.0001), respectively].

The data indicate that VVE extract exhibited antioxidant properties, not only by suppressing intracellular ROS but, additionally, by modulation of the expression of stress-response genes in *C. elegans*, such as *hsp-16.2, sod-3*, and *gst-4*. These abilities were similar to the effects of resveratrol (41), gallic acid (15, 17), catechin (32, 35), and quercetin (15, 24, 42) on oxidative stress resistance in *C. elegans via* the transcription factor *DAF-16/FoxO*. Taken together, the results of this study strongly suggest that the VVE extract mediated antioxidant activity and stress resistance in *C. elegans* via the *DAF-16/FoxO* pathway. However, further studies are required to clarify the underlying mechanisms of the VVE extract on the neuroprotective effect in *C. elegans*.

### Effect of *V. vinifera* Ethanol Extract in Aging

**Figure 7:**
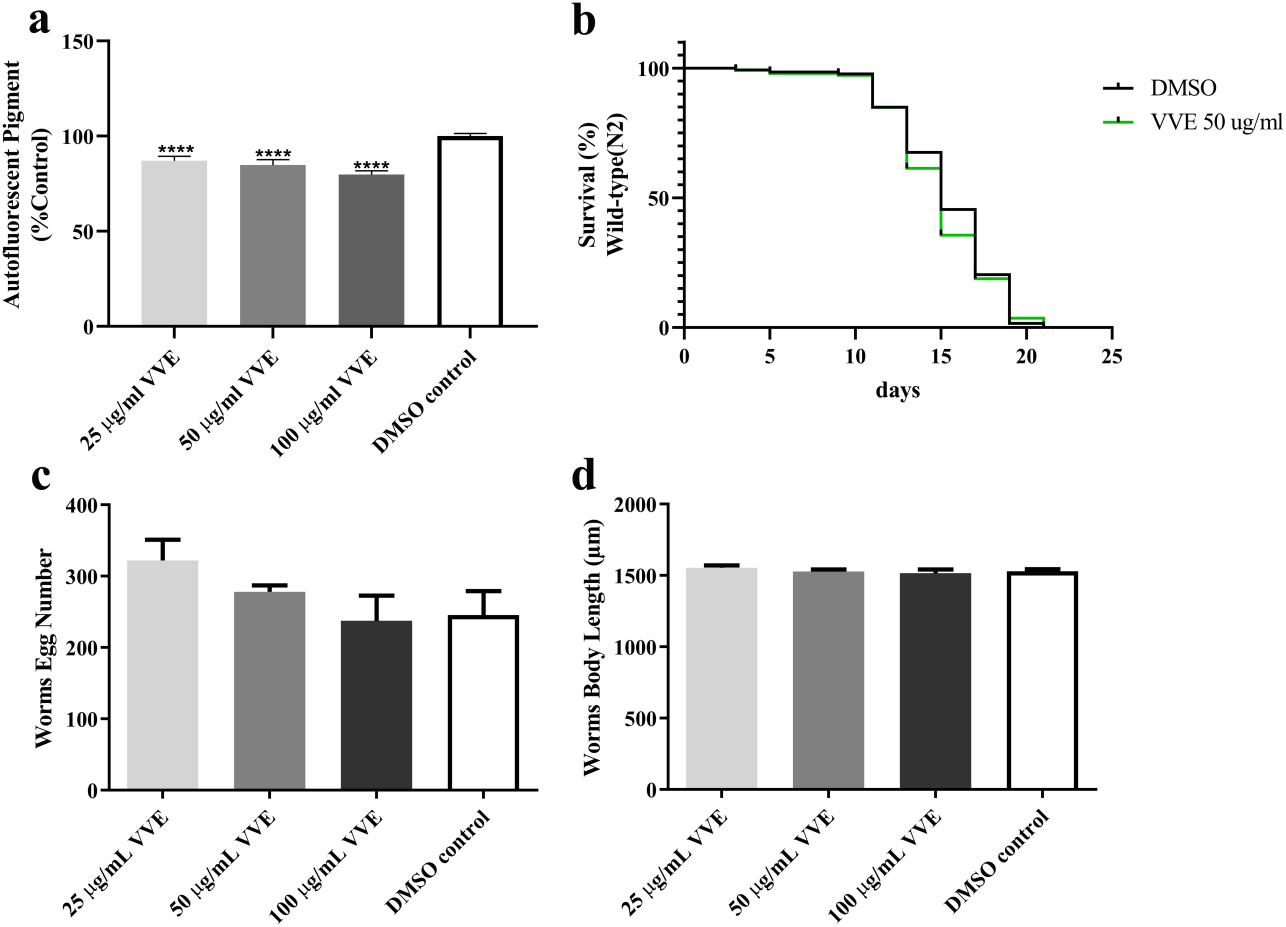
Effect of VVE extracts on aging in *C. elegans*. VVE extracts attenuated autofluorescent pigment in BA17 worms **(a)**. Autofluorescent granules were measured under blue wavelength band. VVE extracts had no effect on life span of wild-type (N2) worms in normal conditions **(b)**. Brood size **(c)** and body length **(d)** of wild-type (N2) worms after VVE extracts treatment. Treatment with VVE extracts had no effect on egg-laying activity and body length. Results are expressed as mean ± SEM from three independent experiments (*n* = 30 worms in each experiment). Treatment groups are compared with DMSO control by one-way ANOVA following Bonferroni's method (*post hoc*).

*C. elegans* is a popular model of aging and longevity (36). Several polyphenols have been reported as antiaging agents in *C. elegans*, such as resveratrol (43), anthocyanin (33), and quercetin (24). To examine the possible influence of VVE extract on aging, the autofluorescent pigment (lipofuscin) accumulation and lifespan were measured. The accumulation of intestinal autofluorescence (lipofuscin) in *C. elegans* during aging is often used as a marker of health or aging (44). We found that the VVE extract significantly decreased the level of lipofuscin accumulation in late adult worms (16 days) (Figure 7a).

Despite the antioxidant capacity *in vitro* and *in vivo* and aging marker reduction, VVE extract did not show any lifespan-prolonging effects in wild-type worms in normal conditions (Figure 7b). These abilities were similar to the effects of resveratrol in the life span of *C. elegans* under normal conditions (43). However, the resveratrol show strongly increased life span effects *in C. elegans* under conditions of oxidative stress (43). Possibly, the antiaging effects of VVE extracts are linked to antioxidant effects. The effects of VVE extract on the life span of *C. elegans* under oxidative stress conditions will be an interesting topic for future study.

To exclude the toxic effect on the reproductive system and dietary restriction system induced by VVE extract, we further measured brood size and body length. Brood size (Figure 7c) and body length (Figure 7d) in wild-type worms were not affected by different concentrations of VVE extract. These data indicated that the effects of VVE extract did not interfere with the fertility rate nor with body development (e.g., *via* dietary restriction) as mentioned in the literature as toxicity markers (33).

## Conclusion

Oxidative stress has been connected to neurodegenerative diseases (1, 2). In this study, HT22 hippocampal neuronal cells and *C. elegans* models were used to study the protective effects of VVE extract against oxidative stress as *in vitro* and *in vivo* studies. We found that the VVE extract protects against glutamate-induced oxidative toxicity in HT22 hippocampal neuronal cells and against juglone-induced oxidative stress in *C. elegans*. The neuroprotective action of the VVE extract in hippocampal neuronal (HT22) cells is mediated *via* inhibition of ROS accumulation and enhancing endogenous antioxidant enzymes. In addition, the VVE extract exhibits oxidative resistance properties in *C. elegans* involved in the *DAF-16/FoxO* signaling pathway. VVE reduced age-related markers (lipofuscin), although it did not extend the life span of *C. elegans* under normal conditions. These studies first report the phytochemical constituents and antioxidant properties of *V.vinifera* leaf extract. The leaf extract might be considered as an alternative supplement or medicine to defend against oxidative stress and neurodegenerative diseases. *In vivo* intervention studies with more complex model organisms are required to support the therapeutic potential of the VVE extract for age-related neurodegenerative disorders.

## Data Availability Statement

The original contributions presented in the study are included in the article/Supplementary Material, further inquiries can be directed to the corresponding authors.

## Author Contributions

CD performed the experiments, analyzed data, and was a major contributor in writing the manuscript. PR performed the gene expression assay by RT-PCR. CD, PR, SZ, and XG designed the study and prepared media and reagents. MW review and editing the manuscript. MW and TT provided materials for the study and conceived and supervised research. MW, TT, PR, SZ, and XG corrected the manuscript. All authors contributed to the article and approved the submitted version.

## Conflict of Interest

The authors declare that the research was conducted in the absence of any commercial or financial relationships that could be construed as a potential conflict of interest.

## Abbreviations

VVE: *Vitis vinifera* ethanol
AD: Alzheimer's disease
DMEM: Dulbecco's modified Eagle's medium
LDH: Lactate dehydrogenase
MTT: 3-(4,5-dimethylthiazol-2-yl)-2,5-diphenyltetrazolium bromide
PCR: polymerase chain reaction
DNA: deoxyribonucleic acid
RNA: ribonucleic acid.
