# Supplementary material for: Vitis Vinifera Leaf Extract Protects Against Glutamate-Induced Oxidative Toxicity in HT22 Hippocampal Neuronal Cells and Increases Stress Resistance Properties in Caenorhabditis Elegans
Source: Front Nutr. 2021 Jun 11;8:634100. doi: 10.3389/fnut.2021.634100 (PMC8225951; doi:10.3389/fnut.2021.634100)
Supplement: Supplementary file 1 [file Data_Sheet_1.PDF]

Supplementary Material

1 Supplementary Materials and Methods

1.1 Qualitative phytochemical screening

The VVE extract was submitted to screen and analyze the phytochemistry using LC-MS (Liquid Chromatography-Mass Spectrometry) at the Institute of Systems Biology (University Kebangsaan Malaysia, Malaysia). The chromatographic separation was carried out on a Dionex™ UltiMate 3000 UHPLC system (Thermo Scientific) equipped with an Acclaim™ Polar Advantage II C18 column (3 × 150 mm, 3 µm particle size) (Thermo Scientific, USA) by using a 1 µL injection volume. The mobile phase comprised 0.1% formic acid in water (solvent A) and 100% acetonitrile (solvent B), which had a flow rate of 400 µL/min for 22 min. At 0-3 min, 3-10 min, 10-15 min, and 15-22 min; 5% B, 80% B, 80% B, and 5% B were used for the gradient elution, respectively. High-resolution MS analysis was carried out in the positive electrospray ionization mode using a MicrOTOF-Q III (Bruker Daltonik GmbH, Bremen, Germany). A capillary voltage of 4500 V, drying gas flow of 8 L/min, an ion source temperature of 200 °C, a nebulizer pressure of 1.2 bar, an end plate offset of −500 V, and a scan range from m/z 50 to 1000 were used as parameters for the instrument. The METLIN and KNApSAcK databases were used for identification of top ten compounds by comparing the observed m/z values with the calculated mass values from previously published data. The abundance of individual compounds was calculated from the percentage of peak area relative to the total area of all peaks in the chromatograms.

Candidate compounds (gallic acid, catechin, epicatechin and quercetin) were characterized and quantified using RP-HPLC analysis at RSU Science and Technology Research Equipment Center (Rangsit University, Thailand)
The chromatography was carried out on SHIMADZU LC-10 HPLC equipped with an analytical C18 reversed-phase column (ODS3 C18, 4.6 × 250 mm i.d., 5-micrometer particle size) and UV detector (best condition at 220 nm). The mobile phase consists of 0.02 M sodium acetate, buffered to a pH of 4 with 0.0125 M citric acid, containing 0.042 M methanesulfonic acid and 0.1 mM EDTA. The flow rate was set at 1 mL/min. The working standard solutions were freshly prepared in 0.05 M perchloric acid containing 0.1 mM Na_2EDTA on ice and stored at −20 °C before using. Peaks were identified by comparing the retention time of each peak in the sample solution, where each individual peak was further compared to the standard solution of gallic acid, catechin, epigallocatechin gallate (EGCG), oxyresveratrol, quercetin, octadecatrienolic acid (linolenic acid), and hexadecanoic (palmitic acid) (Sigma-Aldrich, USA) served as an internal standard. The calibration curves of internal standard compounds were constructed for quantification.

1.2 Determination of cell viability

To perform the MTT assay, after each treatment, the culture medium was added with 0.5 mg/mL MTT and incubated for 3 h at 37 °C. Then, all solution was removed, and the formazan crystals were solubilized by DMSO-ethanol mixture (1:1, v/v). The absorbance at 550 nm was measured using an EnSpire® Multimode Plate Reader (Perkin-Elmer, Waltham, MA, USA). Results were expressed as a percentage relative to the DMSO control.

To assess the LDH assay, the activity of LDH release in culture medium was measured using the CytoTox 96® assay (Promega) according to the manufacturer's instructions. After each treatment, the culture supernatant was incubated with a substrate mix for 30 min in the dark at RT, followed by the addition of a stop solution. The absorbance at 490 nm was read using an EnSpire® Multimode Plate Reader (Perkin-Elmer, Waltham, MA, USA). Results were expressed as a percentage of maximum LDH release obtained by complete cell lysis.

1.3 Measurement of intracellular ROS in HT22 cells

ROS production was quantified by the DCFH-DA method. After treatment, 10 μM H_2DCFDA was added to the culture medium and incubated for 30 min at 37 °C, followed by washing with Hank's balanced salt solution (HBSS). The fluorescence intensity (excitation = 485 nm; emission = 535 nm) was measured using an EnSpire® Multimode Plate Reader (Perkin-Elmer). Data were expressed as the percentage of fluorescence intensity of treated cells relative to the DMSO control.

1.4 RNA isolation and quantitative RT-PCR

The gene specific sequences of primers were SOD1 (Forward: 5'-CAGGACCTCATTTTAATCCTCAC-3', Reverse: 5'- CCCAGGTCTCCAACATGC-3'), CAT (Forward: 5'- CAGCGACCAGATGAAGCA-3', Reverse: 5'- CTCCGGTGGTCAGGACAT-3'), GPx (Forward: 5'- ACAGTCCACCGTGTATGCCTTC-3', Reverse: 5'-CTCTTCATTCTTGCCATTCTCCTG-3'), GSTo1 (Forward: 5'- CAGCGATGTCGGGAGAAT-3', Reverse: 5'- GGCAGAACCTCATGCTGTAGA-3'), GSTa2 (Forward: 5'-TCTGACCCCTTTCCCTCTG-3', Reverse: 5'- GCTGCCAGGATGTAGGAACT-3') and β-actin (Forward: 5'- GGCTGTATTCCCCTCCATCG-3', Reverse: 5'- CCAGTTGGTAACAATGCCATGT-3')

1.5 Brood size and body length assay

L4 larvae of wild-type (N2) worms were sorted and placed one by one on individual NGM agar plates supplemented with different concentrations of VVE extracts and an *E. coli* OP50 lawn as a food source for 24 h. The adult worms were transferred daily to fresh medium to separate them from their progeny. The eggs were counted using a dissecting microscope every day for 4 days to obtain a mean brood size.

To measure the body length, day 1 adult worms were paralyzed using 10 mM sodium azide and mounted on a microscopic glass slide. Photos of worms were photographed using a 10× objective lens of a bright-field microscope. The software BZ-II Analyzer (Keyence Corp.) was used to analyze the body length, which was represented in micrometers.

2 Supplementary Figures and Tables

2.1 Supplementary Figures

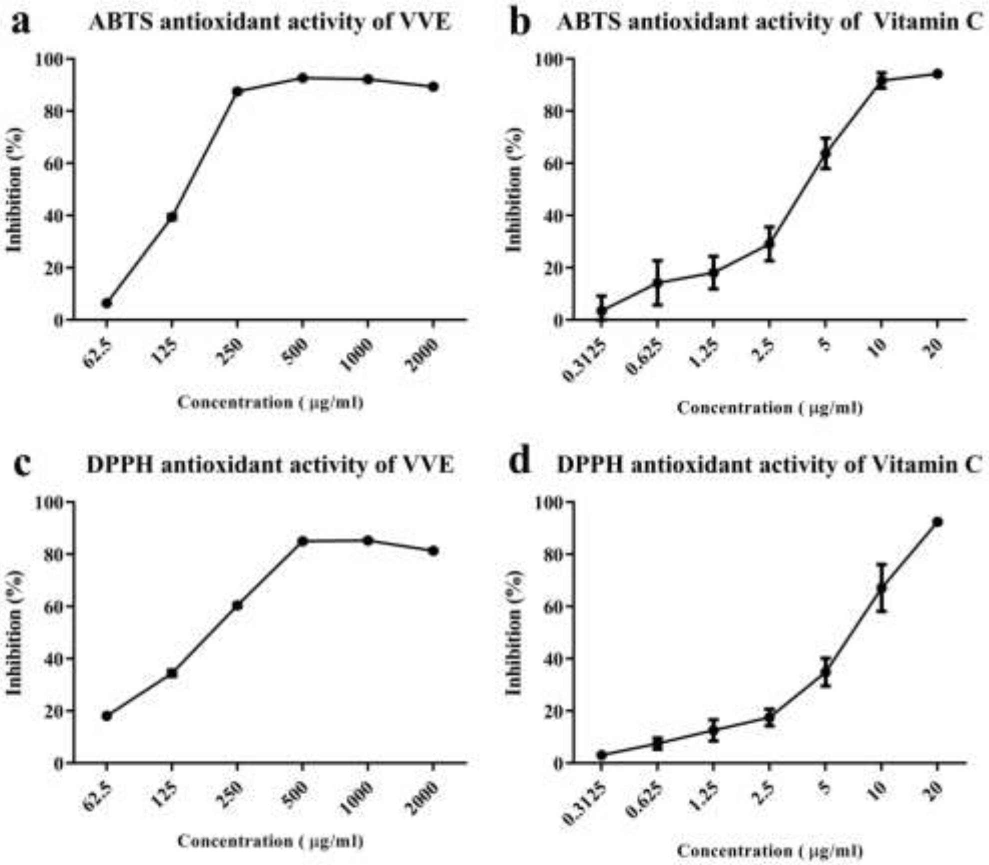

Figure S1. ABTS and DPPH radical scavenging activity of VVE extract (62.5-2000 µg/mL)(a, c), and vitamin C (0.31-20 µg/mL)(b, d).

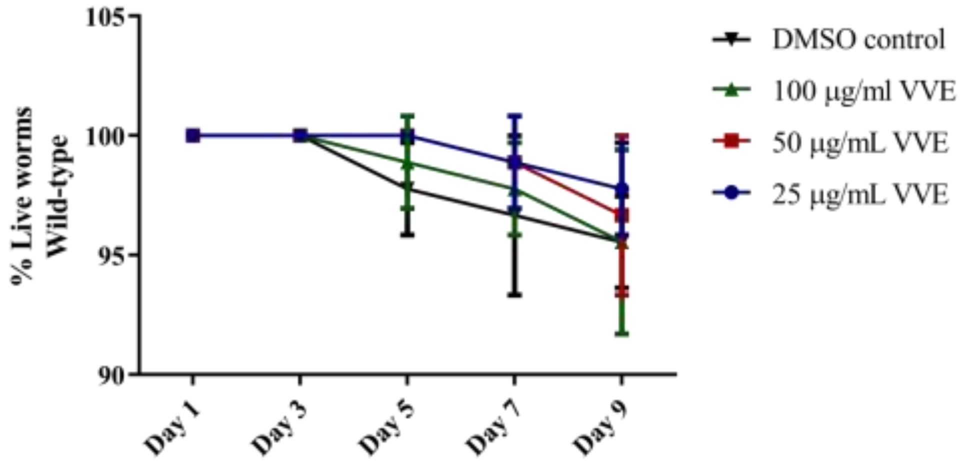

Figure S2. Survival rate of wild-type *C. elegans* after treatment with VVE extracts.

Survival rate of wild-type worms by treatment with different concentrations of VVE extracts for 1-9 days. The results are expressed as the mean ± SEM from three independent experiments (n = 90). Treatment groups are compared to the DMSO control by one-way ANOVA following Bonferroni's method (post hoc).

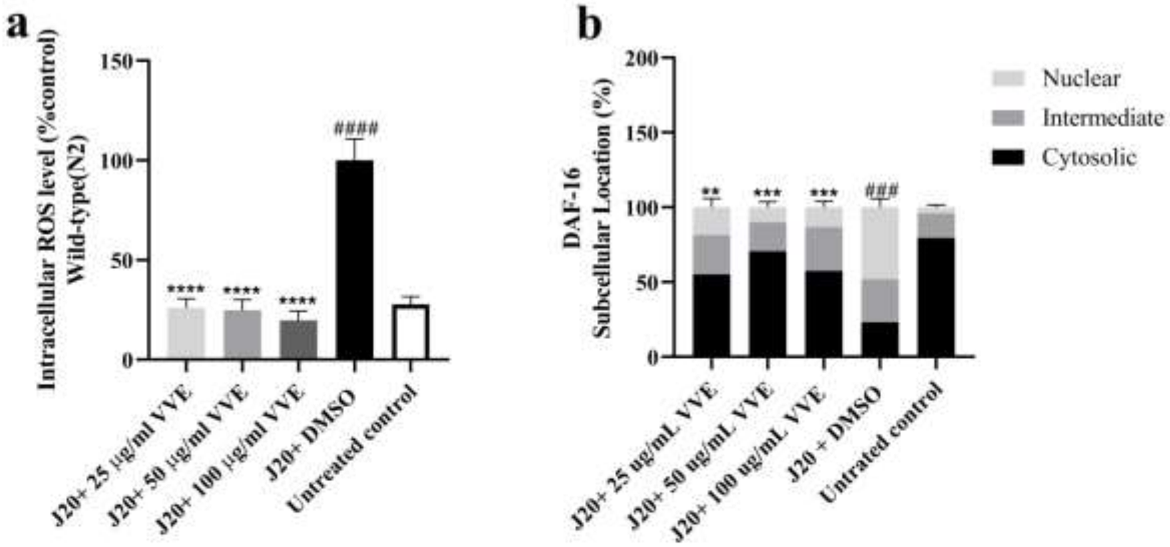

Figure S3. The effects of VVE extracts on intracellular ROS accumulation and DAF-16 translocation under oxidative stress condition in *C elegans*.

Under oxidative stress condition, VVE extracts treatment reduced ROS levels in N2 worms when compared to the juglone treatment group **(a)**. The VVE extracts reduced DAF-16::GFP nuclear translocation in mutant TJ356 worms [daf-16p::daf-16a/b::GFP + rol-6] when compared to the juglone treatment group **(b)**.

Worms were treated with plant extracts for 48 h and exposured to a nonlethal dose of 20 µM juglone (J20) for 24 h. All data are shown as the mean ± SEM of at least three independent experiments. $^{\#\#\#}p < 0.001$, $^{\#\#\#\#}p < 0.0001$ vs. untreated control; $^{*}p < 0.05$, $^{**}p < 0.01$, $^{***}p < 0.001$ and $^{****}p < 0.0001$, compared to the DMSO+ juglone group by one-way ANOVA following Bonferroni's method (posthoc).

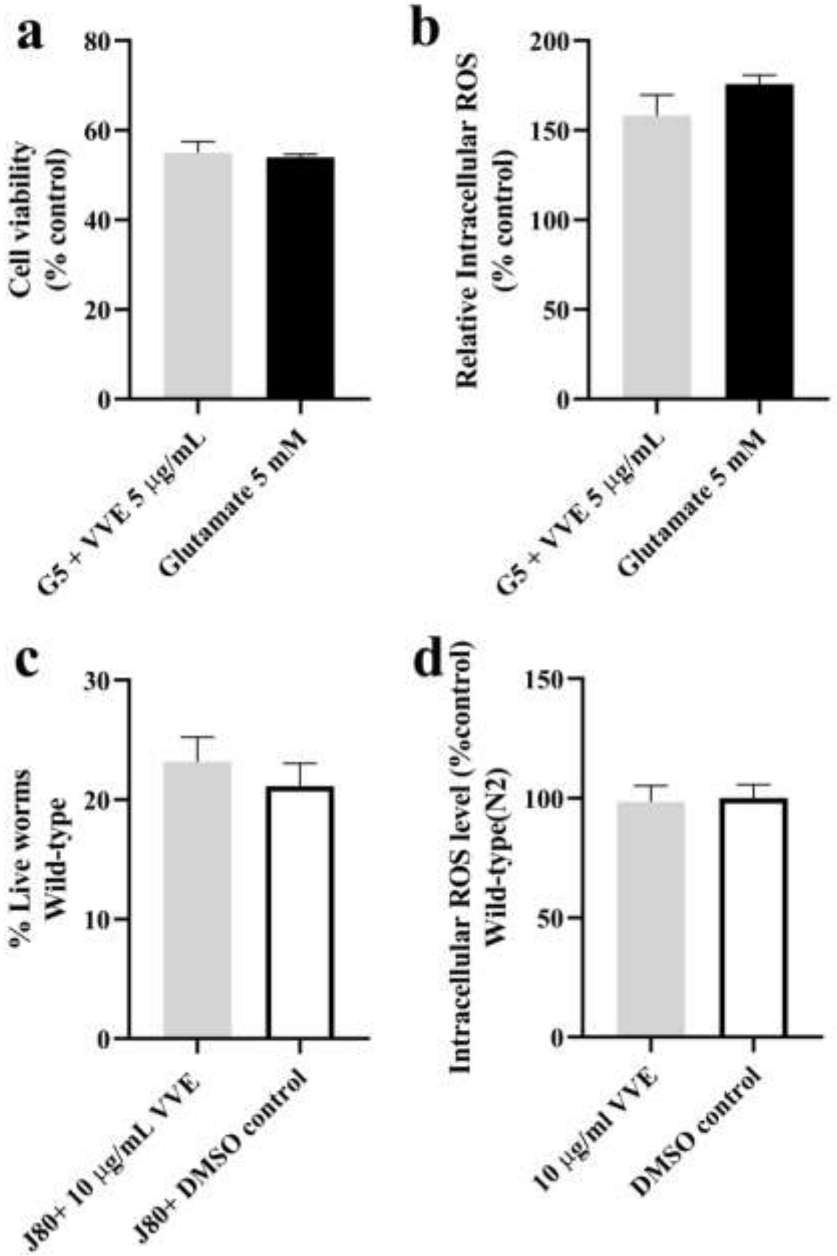

Figure S4. Effect of VVE extracts at low concentrations on the survival rate and the intracellular ROS of HT22 cells and wild-type (N2) worms.

VVE extracts at low concentrations (5 µg/mL in HT22 and 10 µg/mL in N2 worms) failed to neither increase the survival rate (**a**, HT22 cells) (**c**, N2 worms) nor decrease the level of ROS (**b**, HT22 cells) (**d**, N2 worms). DMSO was used as the solvent control. Data are presented as the mean ± SEM (n = 80, replicated three times).

2.2 Supplementary Tables

Table 1. Proposed phytochemical constituents in the VVE extract using LC-MS

Peak No.	Rt (min)	[M + H]+ (m/z)	Area (%)	Proposed compound
46, 47, 48, 49, 50	2.3	229.9793	2.527813	Resveratrol
57	3.1	170.9702	0.092764	Gallic acid
100, 101	6.9	157.9826	9.134053	Apigenin
160	8.6	289.024	0.027543	Catechin
177	9.3	464.9109	1.385883	Quercetin 3-O-glucoside
180, 181	9.3	302.9039	21.8893	Quercetin
208, 209	10	302.9037		
212, 213	10.2	302.9031		
215	10.2	478.8861	3.651668	Quercetin glucuronide
273	13.1	621.0758	7.469807	Tannin

Database: METLIN (CA, USA) and KNApSAcK Keyword Search Web Version 1.000.0

Table 2. Individual phytochemical constituents in the VVE extract using HPLC

Peak No.	Rt (min)	Compound	Concentration*
1	11.7	Gallic acid	18.26
2	21.1	Catechin	55.10
3	24.5	Epicatechin	14.22
4	41.8	Quercetin	197.73

**mg/100 g of crude extract*

Table 3. Total phenolic content, total flavonoid content and free radical scavenging capacity of the VVE extract

	Total Phenolics mgGAE.g **	Total Flavonoids mgQE.g **	DPPH scavenging assay		ABTS scavenging assay	
Extract			%Radical Scavenging activity*	IC_{50} (μg/mL)	%Radical Scavenging activity*	IC_{50} (μg/mL)
VVE extract	62.56 ± 2.25	45.64 ± 0.40	85.31 ± 0.46	249.14 ± 9.6	92.23 ± 2.59	158.75 ± 2.59

*VVE: 1 mg/mL VVE extract, * of 1 mg/mL extract, ** dry weight sample, Values are expressed as the mean ± SD (n = 3)*
